# Aspects épidémiologiques, pronostiques et thérapeutiques de l'hématome retro placentaire (HRP) dans une maternité de référence en zone rurale

**DOI:** 10.11604/pamj.2014.17.11.3554

**Published:** 2014-01-14

**Authors:** Ousmane Thiam, Magatte Mbaye, Abdou Aziz Diouf, Fode Baba Touré, Mamour Gueye, Mansour Niang, Mamadou Lamine Cissé, Sidy Dièye, Jean Charles Moreau

**Affiliations:** 1 Centre Hospitalier Régional, Ndioum, Sénégal; 2 Clinique Gynécologique et Obstétricale, Chu A le Dantec, Dakar, Sénegal

**Keywords:** Hématome rétro placentaire, mortalité maternelle, mortalité périnatale, Retro placental hematoma, maternal mortality, perinatal mortality

## Abstract

**Introduction:**

IL s'agira ici d’ identifier les particularités diagnostiques et la prise en charge de l'HRP au Centre Hospitalier de Ndioum au Sénégal.

**Méthodes:**

Etude rétrospective descriptive menée à l'hôpital régional de Ndioum, durant la période allant du 1er Janvier 2009 au 31 Décembre 2011. Toutes les patientes qui avaient présenté un HRP étaient incluses. Pour chaque patiente, nous avions étudié les paramètres socio-démographiques et cliniques, les données thérapeutiques et le pronostic materno-foetal.

**Résultats:**

La fréquence était de 6,05%. L’âge moyen de nos patientes était compris entre 29 et 84 ans et la parité moyenne de 4,92. Aucune consultation prénatale n’était effectuée chez 16% des patientes. Les évacuations représentaient 66% de cas. L’âge gestationnel moyen était de 36 semaines d'aménorrhées et la majorité des patientes (86%) était en travail. La gravite du tableau clinique était appréciée selon la classification de Sher avec 63% (114 cas) au grade 3. Une coagulopathie était observée chez 27,2% des patientes. Nous avions retrouvé une relation statistiquement significative entre la gravite du tableau clinique et le pronostic maternel (p = 0,026) et foetal (p = 0,0000). Une direction du travail était effectuée chez 109 patientes (60% des cas). L'expulsion foetale était obtenue par voie basse dans 49% des cas et une césarienne était effectuée dans 51% des cas. La morbidité maternelle était dominée par l'anémie secondaire à une hémorragie aigue dans 17,8% des cas et à l'insuffisance rénale aigue dans 6,1% des cas. Les éléments de mauvais pronostic étaient représentés par la gravité du tableau clinique (p = 0,03) et le mode d'admission dominé par les évacuations (p = 0,01), la relation était statistiquement significative. La voie d'accouchement n’était pas retrouvée comme élément de mauvais pronostic (p = 0,09). Nous avions noté douze cas de décès maternels soit 6,6% des patientes.

**Conclusion:**

La prévention de la mortalité maternelle et foetale liée à cette affection passe par une amélioration du suivi prénatal, une meilleure organisation du plan d'accouchement et une amélioration des conditions d’évacuation. La mise en place d'une banque de sang fonctionnel, d'une unité réanimation médicale ainsi qu'un service de prise en charge néonatale devient une nécessité pour améliorer le pronostic maternel et néonatal.

## Introduction

L'hématome retro placentaire (HRP) ou le décollement prématuré du placenta normalement inséré (DPPNI) alors que le foetus est en in utéro. Il conduit à l'interruption des échanges materno-foetales [1–3]. C'est un accident obstétrical imprévisible et grave qui engage le pronostic vital de la mère et celui du foetus. En effet, il nécessite une prise en charge obstétricale doublée d'une réanimation médicale. Sa fréquence est diversement apprécié selon les auteurs: 0,25% en Europe [2, 4, 5], 1 à 9% dans les pays en développement [1, 3].

L'absence de données statistiques sur l'hématome retro placentaire en zone rurale sénégalaise nous a conduit à mener cette étude dont l'objectif est d'identifier les particularités diagnostiques et la prise en charge de l'HRP au Centre Hospitalier de Ndioum au Sénégal.

## Méthodes

### Cadre d’étude

L’étude s'est déroulée au Centre Hospitalier de Ndioum, situé à 500 Km de la capital sénégalaise. La Maternité de l'hôpital couvre 68740 femmes en âge de reproduction (FAR) réparties dans deux districts sanitaires et plusieurs communautés rurales avec 10759 naissances attendues par an. Les activités obstétricales y sont réalisées dans 9 structures de soins obstétricaux d'urgence de base (SOUB). La maternité qui ne dispose que de 42 lits constitue la seule structure offrant des soins obstétricaux et néonatals d'urgence complets (SONUC) disponibles 24heures sur 24. L'hôpital dispose d'un bloc opératoire avec 2 salles fonctionnelles et une banque de sang sans unité de réanimation médico-chirurgicale. La maternité réalise en moyenne 1050 accouchements par an dont un nombre moyen de 300 césariennes soient 28,5% des accouchements.

### Type d’étude

Nous avions mené une étude rétrospective descriptive et analytique durant la période allant du 1er Janvier 2009 au 31 Décembre 2011 soit 3 ans. Toutes les patientes qui avaient présenté un HRP, étaient incluses dans notre étude. Ainsi pour chaque patiente, nous avions étudié les caractéristiques sociodémographiques et cliniques, les données thérapeutiques et le pronostic materno-foetal. La saisie, l'exploitation et l'analyse des données étaient effectuée grâce au logiciel SPSS.

## Résultats

### Fréquence

Durant la période d’étude, 180 cas d'HRP ont été colligés sur total de 2974 accouchements effectués dans le service, soit une fréquence de 6,05%. La moyenne annuelle a été de 60 cas.

### Caractéristiques sociodémographiques

L’âge moyen de nos patientes a été de 29,84 ans avec des extrêmes de 13 et 46 ans. Les patientes âgées de moins de 20 ans étaient retrouvées dans 6% de cas (11) et celles de plus de 35 ans en avaient représenté 31% (56 cas). La parité moyenne a été de 4,92 avec des extrêmes de 1 et 13. Les multipares (4 à 6) et les grandes multipares (≥ 7) représentaient respectivement 25,6% et 37,8% des cas. Les primipares et paucipares étaient de 36,7%. Sur les 180 patientes, 18% avait eu au moins quatre consultations prénatales. Soixante six pour cent avaient eu un nombre de consultation prénatale compris entre un et trois. Cependant 16% des patientes n'avaient effectué aucune consultation prénatale. Sur le plan obstétrical, 12% des patientes avait présenté un antécédent de pré-éclampsie et 11% avait eu un antécédent d'HRP.

### Mode d'admission

Sur les 180 patientes colligées, 119 étaient évacuées d'une autre structure, soit 66% de cas. Celles venues d'elles mêmes étaient de 58 cas soit 32%. Les patientes référées étaient au nombre de trois, soit 2% des cas. La majorité des patientes évacuées venaient du district sanitaire de Podor et repentaient 73% des cas (87 patientes). Les patientes évacuées provenaient pour la majorité des cas d'une distance supérieure à 20 km. L’évacuation de la patiente était effectuée par ambulance dans 83 cas (69%) et, un abord veineux était réalisé dans 91 cas (76%). Le traitement reçu avant l’évacuation consistait à une rupture artificielle des membranes dans 49 cas, une perfusion de macromolécules dans 8 cas et des cristalloïdes dans 27 cas.

### Aspects cliniques et thérapeutiques

L’âge gestationnel moyen était de 36 semaines d'aménorrhée avec des extrêmes de 22 et 40 semaines d'aménorrhée. Plus de 95% des patientes avaient une grossesse comprise entre 7 mois et 9 mois. Pour la majorité des patientes (87%), les signes cliniques à l'admission étaient dominés par les métrorragies (41,7%), la contracture (27,2%), l'absence d'activité cardiaque foetale (21,1%) et l’état de choc (10%). Trente Sept pour cent des patientes présentaient une HTA à l'entrée. Les membranes étaient intactes pour 113 cas, soit 73% des patientes. La majorité (154 cas) des patientes était en période de travail, soient 86% des patientes. Elles étaient à la phase active pour 133 cas (74%) contre 21 cas (12%) à la phase de latence. La gravite du tableau clinique était apprécié selon la classification de Sher, on distingue: grade 1: métrorragies apparemment isolées; grade 2: symptomatologie plus complète et enfant vivant; grade 3: symptomatologie complète avec mort foetale ( 3A: sans troubles de la coagulation;3B: avec troubles de la coagulation). Nous avons retrouvé la prédominance du grade 3 avec 114 cas (63%) (Tableau 1). Une coagulopathie était observé chez 27,2% des patientes.

**Tableau 1:** Répartition des patientes selon le grade de gravité

Grade clinique	Fréquence	Pourcentage
Grade1	4	2,2
Grade 2	62	34,4
Grade 3A	65	36,1
Grade 3B	49	27,2
**Total**	**180**	**100**

Une réanimation basée sur un remplissage vasculaire soit par des macromolécules ou des cristalloïdes étaient effectuées chez toutes les patientes. L'expulsion foetale était obtenue par voie basse chez 88 patientes, soient 49%. La césarienne, dans la moitié des cas (51%) était indiquée d'emblée si l'enfant était vivant ou après un échec à la direction du travail (Tableau 2). Une transfusion de sang était effectuée chez 49 patientes soit 27,2%. Ces patientes avaient reçu en moyenne 1,92 litre de sang total. Cependant pour 94 cas, soit 52,2% le sang était demandé mais non disponible.

**Tableau 2:** Prise en charge des patientes

Rupture des membranes		
Dans la structure	131	73
En dehors de la structure	49	27
**Direction du travail**		
Oui	109	60
Non	71	40
**Accouchement**		
Voie Basse	88	49
Césarienne	92	51
**Transfusion**		
Oui	131	27.2
Non	49	72.7

Le délai moyen entre admission et accouchement a était de 1,95 heure (117min) avec des extrêmes de 10min et de 16 heures (960min). La majorité des patientes avait effectué une expulsion avant les 3heures. Le poids moyen des caillots a était de 363,17 grammes avec une expulsion maximale de 1100 grammes de caillots.

### Le devenir maternel et foetal

#### Le devenir maternel

La morbidité maternelle a été dominée par l'anémie secondaire à l'hémorragie dans 32 cas, soient 17,8%. Un tableau d'insuffisance rénale aigue était observé dans 11 cas (6,1%) et une association éclampsie et HRP dans trois cas représentant 1,7% des cas. Les éléments de mauvais pronostic maternel étaient représentés par la gravité du tableau clinique d'où on notait 63% de grade 3 avec une relation statistiquement significative (p = 0,03), le mode d'admission dominait par les évacuations qui représentait 66% des patientes avec une relation statistiquement significative (p = 0,01). Cependant la voie d'accouchement n’était pas retrouvé comme élément de mauvais pronostic, la relation statistique n’était pas significative (p = 0,09). Nous avions noté douze cas de décès maternel soit une létalité globale de 6,6%. Parmi elles, 5 femmes avaient accouché par voie basse, soit 41,6%; et 7 par césarienne, soit 58,4%. Ces décès étaient survenus après l’évacuation utérine et la coagulopathie était responsable dans les 7 cas, l'anémie par hémorragies aigues du post-partum était la cause de décès chez 5 patiente (2,7%).

#### Le devenir foetal

Nous avions enregistré 109 décès foetaux et 71 nouveau-nés vivants. La mortalité périnatale était de 60,5%. Le poids moyens de naissance était de 2142 grammes avec des extrêmes de 500 grammes et 4500 grammes. Un faible poids de naissance était observé chez 108 patientes, soient 60% des cas. Les facteurs de mauvais pronostic foetal étaient représentaient par la gravite du tableau avec une relation statistiquement significative (p = 0,000), le faible poids de naissance (p = 0,02), la voie d'accouchement (p = 0,03) et l’évacuation sanitaire (p = 0,001).

## Discussion

### Aspects épidémiologiques et cliniques

La fréquence de l'hématome retro placentaire dans notre série était de 6% alors que Sarr [5] au CHU de Dakar la retrouvait à 2,97%. Dumont [3] à Saint louis rapporte une incidence de 4,2%. Cependant Thieba [6] lui rapporte une incidence de 0,96%. Les séries occidentales avancent des taux qui varient entre 0,4 et 1% [1, 4]. Au Centre Hospitalier de Ndioum, l’âge moyen de nos patientes présentant un HRP était de 29 ans; la parité moyenne à 4,9 et évacuée dans la majorité des cas. Ces constations sont corroborées par les séries africaines [5–8].

Dans notre série, 16% des patientes n'avaient bénéficiées d'aucune consultation prénatale. Cette donnée est comparable à celles rapportées par Gaye à Baudouin [7] et Diallo au CHU de Dakar [7] qui retrouvaient respectivement 16% et 21,4%.

L’évacuation sanitaire était le mode d'admission dominant dans notre série (66%). Ce taux est relativement inferieur à ceux rapportés par Thieba à Ouagadougou [6], Diallo à Dakar [8] et Touré à Abidjan [9], qui retrouvaient respectivement 89%, 80,1% et 81,9%. Ce mode d'admission était retrouvé dans notre série comme un facteur de mauvais pronostic maternel et foetal.

Le délai moyen entre l'admission et l'accouchement était de 1,95 heures. Ce délai retrouvait dans notre série est nettement inferieure à celui rapporté dans la littérature. Le recours à la césarienne était de 51%, taux nettement plus élevé à celui enregistré au CHU de Dakar [5] qui était chiffrait à 2,25% tandis que Gaye à Baudouin [8] et Thieba [6] à Ouagadougou avaient respectivement rapporté 15% et 35,6%. Notre taux se rapproche de celui des données occidentales qui oscille entre 50% et 100% [1, 10]. L'insuffisance des moyens de réanimation notamment les difficultés de compenser la spoliation sanguine importante et prolongée, aggravent le pronostic de ces jeunes multipares évacuées souvent dans de mauvaises conditions et présentant à l'admission un tableau clinique grave. Ce constat nous conduit à réduire le délai et ainsi faire recours à la prise en charge chirurgicale.

### Aspects pronostiques et thérapeutiques

Dans notre série la létalité maternelle de l'hématome retro placentaire se situe autour de 6,6%. Ce taux reste comparable aux données des pays en développement [1, 10] mais reste supérieur à ceux rapportés dans la littérature Occidentales [1, 9]. Toutes fois, si nous tenons compte de la place de l'anémie et de la Coagulation Intra vasculaire disséminée dans la mortalité dans notre série, l'amélioration de la létalité passe par la transfusion sanguine. Le taux de transfusion sanguine dans notre étude (27% des cas) était comparable à celui de Dumont [3] à Saint Louis qui rapporte 21%, supérieur à celui de Gaye [8] à Baudouin qui rapporte 18% et nettement inférieur à celui de Diallo au CHU de Dakar [5] estimé à 33,7%.

Les principales causes de morbidité que nous avions retrouvées étaient en rapport avec l'anémie et l'insuffisance rénale aigue, confirmant ainsi les données de la littérature africaine [2, 3, 8, 11].

La mortalité périnatale dans notre étude se situait au tour de 60%, en rapport avec la gravité du tableau clinique mais également avec l'importance du taux de faible poids de naissance (60,5%), dans une maternité dépourvue d'unité de réanimation néonatale. Le foetus paie ainsi le plus lourd tribut de cette pathologie. Ce taux est nettement inférieur à ceux rapportés dans les pays en voie de développement qui se situe au tour de 80% [3, 4, 8, 10], mais nettement supérieur au données de la littérature Occidentale [1, 9, 12] où les chiffres sont exceptionnellement supérieur à 20%.

## Conclusion

L'hématome retro placentaire demeure une urgence médico-obstétricale majeure dans notre pratique. La prévention de la mortalité maternelle et foetale liée à cette affection passe par une amélioration de la consultation prénatale, une meilleure organisation du plan d'accouchement surtout en maternité périphérique et une amélioration des conditions d’évacuation surtout en zone rurale ou les moyens sont très réduits. Au niveau de la structure de référence, la mise en place d'une banque de sang fonctionnel, d'unité réanimation médicale adaptée ainsi qu'un service de prise en charge néonatale devient une nécessité afin d'améliorer le pronostic maternel et néonatal.
